# Vitamin D Deficiency and Hashimoto’s Thyroiditis in Children and Adolescents: a Critical Vitamin D Level for This Association?

**DOI:** 10.4274/jcrpe.2011

**Published:** 2015-06-03

**Authors:** Olcay Evliyaoğlu, Manolya Acar, Bahar Özcabı, Ethem Erginöz, Feride Bucak, Oya Ercan, Mine Kucur

**Affiliations:** 1 İstanbul University Cerrahpaşa Faculty of Medicine, Department of Pediatric Endocrinology, İstanbul, Turkey; 2 İstanbul University Cerrahpaşa Faculty of Medicine, Department of Public Health, İstanbul, Turkey; 3 İstanbul University Cerrahpaşa Faculty of Medicine, Department of Biochemistry, İstanbul, Turkey

**Keywords:** Hashimoto thyroiditis, Vitamin D, deficiency, children, adolescents

## Abstract

**Objective:**

Vitamin D has been suggested to be active as an immunomodulator in autoimmune diseases such as Hashimoto’s thyroiditis (HT). The goal of the present study was to investigate the vitamin D status in HT patients.

**Methods:**

This prevalence case-control study was conducted on 90 patients with HT (of ages 12.32±2.87 years) and 79 age-matched healthy controls (11.85±2.28 years). Serum 25-hydroxyvitamin D3 [25(OH)D3] levels were measured in all 169 subjects.

**Results:**

The prevalence of vitamin D deficiency in HT patients (64 of 90; 71.1%) was significantly higher than that in the control group (41 of 79; 51.9%) (p=0.025). Mean serum 25(OH)D3 level in the HT group was significantly lower compared to the control group (16.67±11.65 vs. 20.99±9.86 ng/mL, p=0.001). HT was observed 2.28 times more frequently in individuals with 25(OH)D3 levels <20 ng/mL (OR: 2.28, CI: 1.21-4.3).

**Conclusion:**

Vitamin D deficiency is associated with HT in children and adolescents. Levels lower than 20 ng/mL seem to be critical. The mechanism for this association is not clear.

## INTRODUCTION

Vitamin D is a secosteroid hormone which is activated by enzymes and acts via receptors. In the liver, vitamin D is hydroxylated to 25-hydroxyvitamin D3 [25(OH)D3], which is the major circulating and stored form of the hormone and also the form that is measured to determine vitamin D status. A further hydroxylation step is required in the kidneys to convert 25(OH)D3 into the biologically active form of 1.25-dihydroxyvitamin D [1.25(OH)2D3] (1,2).

1.25(OH)2D3 interacts with its vitamin D nuclear receptor which is present in the small intestine, kidneys and other tissues (3,4). In addition to its central role in calcium and bone metabolism, 1.25(OH)2D3 has important effects on the growth and differentiation of many cell types and also on immune regulation (5,6). Low 25(OH)D3 levels appear to increase susceptibility to immune-related disorders such as autoimmune diseases, tuberculosis and cancers. Furthermore, vitamin D deficiency has been suggested to have an active role in the pathogenesis of autoimmune diseases (7,8,9).

There are various experimental models studying the immunomodulatory effects of 1.25(OH)2D3 and its analogues in autoimmune disease. It has been reported that in lpr/lpr mice systemic lupus erythematosus (10), experimental allergic encephalomyelitis (11,12,13), collagen-induced arthritis (14), Lyme disease (15), inflammatory bowel disease (16) and autoimmune diabetes in nonobese diabetic mice could be prevented by administration of 1.25(OH)2D3 and its analogues (17). In experimental models of autoimmune thyroiditis, the additive protective effect of cyclosporin A and 1.25(OH)2D3 administration has been shown (18,19). In the clinical context, it has been postulated that vitamin D deficiency might increase the risk of autoimmune diseases, including Hashimoto’s thyroiditis (HT) (1,2,5,20).

In this study, we aimed to determine if HT patients were vitamin D-deficient by comparing their serum vitamin D levels with those of healthy controls.

## METHODS

### Patients and Controls

The serum vitamin D levels of 90 (67 females and 23 males) newly diagnosed HT patients between ages 3.64 and 17.16 years (mean: 12.32±2.87) who had presented to the Cerrahpaşa Faculty of Medicine Pediatric Endocrinology Clinic were compared with those of an age-and sex-matched healthy control group (n=79) (Table 1). The control group consisted of children who had applied to the general pediatric outpatient clinic of the same hospital with complaints of mild upper respiratory tract infection. The blood samples were drawn between February 2013 and June 2014. In İstanbul, the weather during the months from April to October is quite clear, thus these months were assumed to represent months with enough sun exposure. In the study period of 17 months, 10 months were with enough sun exposure and the subjects in both groups were chosen to be in similar numbers in the same time interval, thus similar with regard to sun exposure. A chi-square test was performed which confirmed that the inviduals with HT and those in the control group were distributed homogenously.

None of the patients had been treated with L-thyroxine (LT4) at the time the samples were taken. The diagnosis of HT was based on the following findings: anti thyroid peroxidase (anti-TPO) levels >35 IU/mL, anti-thyroglobulin (anti-Tg) levels >40 IU/mL and parenchymal heterogeneity on thyroid ultrasound. Patients with renal and liver disorders, diabetes mellitus, metabolic bone and parathyroid disorders or epilepsy treated by anticonvulsant therapy and patients on other medications that may alter 25(OH)D3 or 1.25(OH)2D3 metabolism and thyroid functions were excluded. The study was conducted between February 2013 and June 2014 and approved by the Cerrahpaşa Faculty of Medicine Ethics Committee (approval date and number: 2-12-2013/3518). Written informed consent was taken from all parents and from children older than 16 years.

Overt hypothyroidism (OHP) was diagnosed if serum thyroid stimulating hormone (TSH) was >5.7 µU/mL (reference interval: 0.7-5.7 µU/mL) and free T4 (fT4) was <0.7 ng/dL (reference interval: 0.7-1.9 ng/dL). Subclinical hypothyroidism was diagnosed if the serum TSH level was increased (>5.7 µU/mL), but serum fT4 and free triiodothyronine (fT3) (reference interval: 1.8-4.2 pg/mL) levels were within the reference ranges. Overt hyperthyroidism was diagnosed if serum TSH was <0.7 µU/mL and fT4 >1.9 ng/dL. Subclinical hyperthyroidism was diagnosed if serum TSH level was <0.7 µU/mL and serum fT4 and fT3 levels were within normal limits. The patients were considered to be euthyroid if their serum fT4 and TSH levels were within normal limits.

Serum 25(OH)D3 levels ≤20 ng/mL, those between 21-29 ng/mL and ≥30 ng/ mL were accepted as vitamin D deficiency, insufficiency and sufficiency, respectively (21). Thus, patients and controls were grouped as 25(OH)D3 deficient, insufficient and sufficient. Patients with 25(OH)D3 deficiency, insufficiency and sufficiency were compared according to their serum TSH, fT3, fT4, total T3, total T4, anti-TPO and anti-Tg levels.

### Laboratory Measurements

Serum TSH, total T3, total T4, fT3, fT4, anti-TPO, anti-Tg and 25(OH)D3 levels were measured in venous blood samples. TSH, total and fT4, total and f T3, anti-TPO and anti-Tg were measured by chemiluminescence method using the autoanalyzer Architect i2000sr (Abbott Laboratories, IL USA).

For measuring 25(OH)D3, venous blood samples were collected into plain tubes, serum was separated and stored at -70 ˚C for a week until analysis. 25(OH)D3 vitamin levels were measured by the HPLC method using a Shimadzu analyzer (Shimadzu Corporation Kyoto, Tokyo, Japan). The intra- and inter-assay coefficients of variation for 25(OH)D3 were 5.6 and 8.0, respectively. The lower detection for 25(OH)D3 was 4 ng/mL (10 mmol/L). No external standards were used.

### Statistics

All statistical analysis were performed by using the software SPSS for Windows V16.0. Vitamin D level distribution was compared with chi-square test between patients and the control group. Seasonal homogeneity of the drawn blood samples of the individuals with HT and the controls were confirmed by chi-square test. Odd ratios between HT and serum 25(OH)D3 levels were calculated by using one variable logistic regression. Comparing means between groups of more than 2 were done by using the Kruskal-Wallis method. Comparison between 2 groups was performed by Mann-Whitney U-test. By using Bonferroni correction, a p-value of 0.016 was accepted as significant between 2 groups. Nonparametric correlations were performed by using Spearman’s test. The data are expressed as means ± standard deviation. A p-value below 0.05 was considered as statistically significant.

## RESULTS

**Figure 1:**
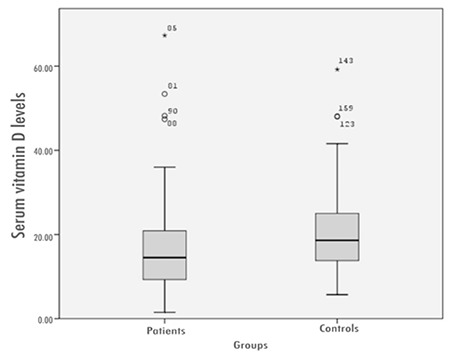
Serum 25-hydroxyvitamin D3 levels in Hashimoto’s thyroiditis patients and in the controls.

**Figure 2:**
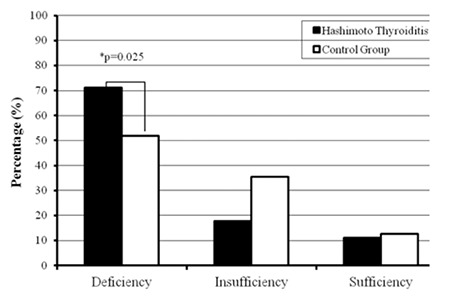
Prevalence of 25-hydroxyvitamin D3 [25(OH)D3] deficiency, insufficiency and sufficiency in Hashimoto thyroiditis patients and in the control group. Prevalence of 25(OH)D3 deficiency in Hashimoto thyroiditis patients was significantly higher than that of the control group (*p=0.025).

Demographic characteristics of the patients with HT and the control group are presented in Table 1. Median 25(OH)D3 level was 16.67 ng/mL (range: 1.5-67.30 ng/mL) for patients with HT and 20.99 ng/mL (range: 5.7-59.20 ng/mL) for the controls (Figure 1). Mean serum 25(OH)D3 level was 16.67±11.65 ng/mL and 20.99±9.86 ng/mL in patients with HT and in the controls, respectively. Mean 25(OH)D3 level was significantly lower in HT patients as compared with the healthy control group (p=0.001). To further evaluate the relationship between 25(OH)D3 levels and HT, the odd ratio between 25(OH)D3 levels and HT was calculated by one variable logistic regression. HT was observed to be more frequent in the individuals with a 25(OH)D3 level <20 ng/mL (OR: 2.28, CI: 1.21-4.3). In patients with HT, the prevalence rates of 25(OH)D3 deficiency, insufficiency and sufficiency were 71% (n=64), 17.7% (n=16) and 11.1% (n=10), respectively. In the control group, these prevalence figures were 51.9% (n=41), 35.4% (n=28) and 12.6% (n=10) in the same order. In HT patients, the incidence of 25(OH)D3 deficiency was significantly higher than that of the control group (p=0.025) (Figure 2).

Euthyroidism, subclinical hypothyroidism, OHP and subclinical hyperthyroidism were diagnosed in 51 (56.6%), 30 (33.3%), 7 (7.77%) and 2 (2.2%) of the patients. None of the patients had overt hyperthyroidism. Because of the low numbers, patients with OHP and subclinical hyperthyroidism were omitted from statistical analysis. Patients with subclinical hypothyroidism and euthyroidism were similar in terms of age and sex distribution. 25(OH)D3 levels were not different among patients with euthyroidism and subclinical hypothyroidism. 25(OH)D3 levels of the euthyroid patients were significantly lower than those of the control group (p=0.005) (Table 2).

Table 3 shows all patients with HT grouped according to their 25(OH)D3 levels. Between groups, serum TSH was the parameter which showed significant differences (p=0.029). Serum TSH level was significantly higher in the patients with 25(OH)D3 deficiency as compared to patients with insufficiency (p=0.011). Thyroid hormone and autoantibody levels showed no differences between the groups.

**Figure 3:**
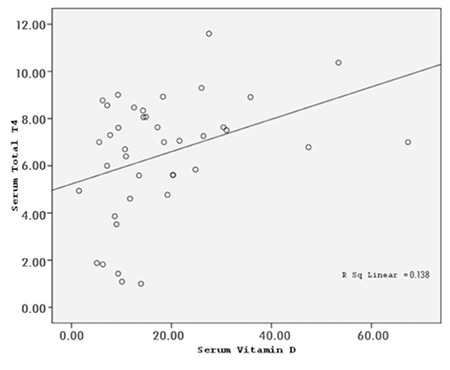
There was a positive correlation between serum 25-hydroxyvitamin D3 levels and total thyroxine in the patients with autoimmune thyroiditis (r=0.37, p<0.05).

In the total group of patients, serum 25(OH)D3 level was positively correlated with serum total T4 (r=0.37; p=0.018) (Figure 3). There was no correlation between 25(OH)D3 levels and T3, TSH or thyroid autoantibodies.

## DISCUSSION

The results of the present study demonstrated that patients with HT had significantly lower serum 25(OH)D3 levels compared with healthy controls and that the prevalence of 25(OH)D3 deficiency was higher in HT subjects. Recently, the effects of vitamin D on autoimmune diseases have come to attention. While some clinical studies have shown a relationship between autoimmune thyroid diseases and serum vitamin D levels, others have not. In a study on 161 adult patients with HT, 25(OH)D3 levels were reported to be lower and prevalence of 25(OH)D3 insufficiency (<30 ng/mL) to be significantly higher in the patients than the 162 controls (22). In a study that involved 78 children (mean age: 12.0±2.8 years) with HT, 25(OH)D3 deficiency (5-14.9 ng/mL) and severe deficiency (<5 ng/mL) rates were higher than the control group (n=74) and an inverse correlation between vitamin D and anti-TPO levels were reported (20). Prevalence of 25(OH)D3 deficiency (<10 ng/mL) was also reported to be higher in adult patients with autoimmune thyroid disease (n=50) compared with patients with non-autoimmune thyroid disease and with healthy controls (23).

Moreover, in adult patients with HT, newly diagnosed and euthyroid both with no treatment (n=180) and in those previously diagnosed and on LT4 treatment (n=180), serum 25(OH)D3 levels were found to be lower than those in the control group (n=180), while 25(OH) D3 deficiency severity was correlated with duration of HT, thyroid volume and antibody levels (24). In a study that involved 6685 men, pre- and post-menopausal women, only in pre-menopausal women, mean serum 25(OH)D3 level was low in subjects with HT (25). In contrast, in a study with two case-control groups, serum 25(OH)D3 levels were not different in adult subjects with genetic susceptibility and in newly diagnosed thyroid autoimmunity compared to healthy controls (26). Additionally, in a study that involved 642 subjects between ages 16 and 60 years, mean serum 25(OH) D3 level was not different in anti-TPO positive and negative individuals (27).

These conflicting results can be partly related to the variations in study designs and in the definitions of vitamin D deficiency and in a lack of consensus regarding optimal serum 25(OH)D3 levels among healthy subjects. Thus, it is not easy to draw a conclusion about whether vitamin D deficiency is a risk factor for the development of autoimmune thyroiditis.

From a different point of view, it has been postulated that the low levels of serum 25(OH)D3 seen in autoimmune disease is a secondary phenomenon which reflects down regulation of vitamin D metabolism rather than being a causal factor leading to illness. Vitamin D receptor (VDR) dysfunction is proposed to be the key factor for the disease process (28). Once 1.25(OH)2D3 activates its receptor, expression of more than 900 genes is affected (29,30). One of the most important effects of VDR activation is its ability to increase the innate immune response which is important in the pathogenesis of autoimmune diseases (29,31,32). It has been postulated that low 25(OH)D3 is the result of VDR dysfunction and not the reason for autoimmunity. In this model, if VDR is disabled by disease, it becomes unable to express CYP24A1 which is the enzyme primarly responsible for inactivating 1.25(OH)2D3. Increased 1.25(OH)2D3 will in turn decrease 25(OH)D3 levels by reducing gene expression by the pregnane X nuclear receptor and inhibiting expression of CYP27A1 which is an enzyme involved in conversion of vitamin D into 25(OH)D3 (33). In a clinical study focusing on VDR in 111 patients with HT, VDR gene TaqI TT and FokI FF genotypes were associated with increased risk for HT (34). Vitamin D levels of these patients were not measured, thus the association between VDR polymorphism and vitamin D status was not determined.

In the current study, although the prevalence rates of 25(OH)D3 insufficiency and sufficiency were not different in patients and controls, the prevalence of 25(OH)D3 deficiency was significantly higher in the HT patients. It was interesting to note that although statistically insignificant, the incidence of 25(OH)D3 insufficiency was higher in the control group. Vitamin D insufficiency was also encountered in our control group, but the patients with HT had vitamin D levels even lower. Based on the results of our study, we suggest that a serum 25(OH)D3 level of 20 ng/mL can may be accepted as a critical cut-off level.

Thus in individuals with serum 25(OH)D3 <20 ng/mL, HT was observed 2.28 times more frequently as compared to ones with higher 25(OH)D3 levels. A recent study evaluating the effects of vitamin D in immune modulation showed a direct correlation of vitamin D levels with T regulatory cells and an inverse correlation with T effector cells, which are both related in the pathogenesis of autoimmune diseases. These authors suggested that there is an optimal vitamin D level to control immune regulation and pathogenic resistance (35). Clinical studies also suggested a U-shaped relation between vitamin D levels and different disorders so that both low and high levels were associated with the diseases (36,37,38,39,40,41). Although in the present study we were unable to evaluate high 25(OH)D3 levels because of the low numbers of patients and controls, our results do support the hypothesis that HT is associated with 25(OH)D3 levels below or equal to 20 ng/mL.

Besides affecting the thyroid gland through immune-mediated processes, vitamin D has also been shown to affect thyroid functions. In rat thyroid cells, vitamin D attenuates TSH-stimulated iodide uptake and cell growth (42). Vitamin D modulates TSH secretion of pituitary thyrotrophs by binding to specific binding sites (43). In a study on 2582 subjects between ages 15 and 98 years, it was found that lower 25(OH)D3 levels were associated with higher TSH levels, independent of age, sex, BMI, serum anti-TPO and/or anti-Tg in the subjects between 15-44 ages (44). In this study, mean serum TSH level was higher in patients with 25(OH)D3 deficiency compared to the patients with insufficiency and in all of the patients there was a positive correlation between serum total T4 and 25(OH)D3 levels. However, 25(OH)D3 levels were not different in patients with subclinical hypothyroidism and euthyroidism. Additionally, 25(OH)D3 levels were not different in patients with subclinical hypothyroidism and in the control group, whereas 25(OH)D3 levels were lower in euthyroid patients compared to the control group. Due to these contradictory results regarding the relationship between serum 25(OH)D3 levels and thyroid functions, it is difficult to make a clear association between them. Although there are studies showing correlation between serum 25(OH)D3 and anti-TPO levels (20,23) we were not able to find any correlation between these two parameters.

In conclusion, this study showed that possibly there is an association between vitamin D and HT, especially in individuals with 25(OH)D3 levels lower than 20 ng/mL. The mechanism for this association and whether it is a cause or effect relationship is not clear.

## Figures and Tables

**Table 1:**
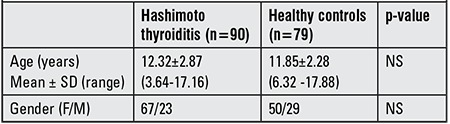
Demographic characteristics and 25-hydroxyvitamin D3 levels of patients with Hashimoto’s thyroiditis and of the healthy control group.

**Table 2:**
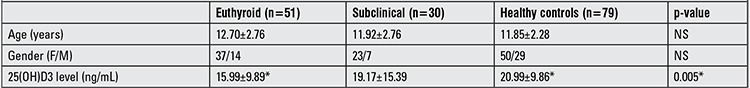
Characteristics and serum 25-hydroxyvitamin D3 [25(OH)D3] levels of patients with euthyroid Hashimoto’s thyroiditis and subclinical hypothyroidism versus healthy controls.

**Table 3:**
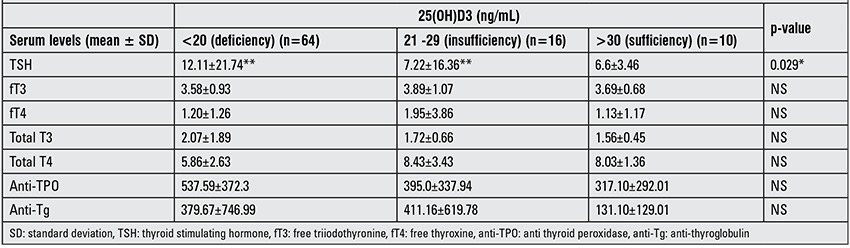
Comparison of thyroid function tests and thyroid autoantibodies of the patients at admission according to their 25-hydroxyvitamin D3[25(OH)D3] status.
